# Therapeutic Targeting of Hyaluronan in the Tumor Stroma

**DOI:** 10.3390/cancers4030873

**Published:** 2012-09-06

**Authors:** Anne Kultti, Xiaoming Li, Ping Jiang, Curtis B. Thompson, Gregory I. Frost, H. Michael Shepard

**Affiliations:** 1 Department of Research, Halozyme Therapeutics, 11388 Sorrento Valley Road, San Diego, CA 92121, USA; E-Mails: mshepard@halozyme.com (H.M.S.); 2 Department of Pharmacology and Safety Assessment, Halozyme Therapeutics, 11388 Sorrento Valley Road, San Diego, CA 92121, USA; E-Mails: xli@halozyme.com (X.L.); pjiang@halozyme.com (P.J.); cthompson@halozyme.com (C.B.T.); 3 Department of General and Administrative, Halozyme Therapeutics, 11388 Sorrento Valley Road, San Diego, CA 92121, USA; E-Mail: gfrost@halozyme.com (G.I.F.)

**Keywords:** cancer, tumor stroma, hyaluronan, hyaluronidase, PEGPH20

## Abstract

The tumor stroma, consisting of non-malignant cells and the extracellular matrix, undergoes significant quantitative and qualitative changes throughout malignant transformation and tumor progression. With increasing recognition of the role of the tumor microenvironment in disease progression, stromal components of the tumor have become attractive targets for therapeutic intervention. Stromal accumulation of the glycosaminoglycan hyaluronan occurs in many tumor types and is frequently associated with a negative disease prognosis. Hyaluronan interacts with other extracellular molecules as well as cellular receptors to form a complex interaction network influencing physicochemical properties, signal transduction, and biological behavior of cancer cells. In preclinical animal models, enzymatic removal of hyaluronan is associated with remodeling of the tumor stroma, reduction of tumor interstitial fluid pressure, expansion of tumor blood vessels and facilitated delivery of chemotherapy. This leads to inhibition of tumor growth and increased survival. Current evidence shows that abnormal accumulation of hyaluronan may be an important stromal target for cancer therapy. In this review we highlight the role of hyaluronan and hyaluronan-mediated interactions in cancer, and discuss historical and recent data on hyaluronidase-based therapies and the effect of hyaluronan removal on tumor growth.

## Abbreviation

BRCA1: breast cancer 1
BSA: bovine serum albumin
CD44: cluster of differentiation 44
Col1α1: collagen, type 1, alpha 1
Col5α1: collagen, type 5, alpha 1
CS: chondroitin sulfate
DS: dermatan sulfate
ECM: extracellular matrix
EGF: epidermal growth factor
ERK1/2: extracellular signal-regulated kinase 1/2
ERM: ezrin-radixin-moesin
FAK: focal adhesion kinase
GFP: green fluorescent protein
HABP: hyaluronan binding protein
GPI: glycosylphosphatidylinositol
GTPase: guanosine triphosphatase
HA: hyaluronan
HAS: hyaluronan synthase
hdf: heart defect
HER: human epidermal growth factor receptor
HS: heparan sulfate
HYAL: hyaluronidase
HYALP-1: hyaluronidase pseudogene 1
IαI: inter-α-inhibitor
KPC: LSL-Kras^G12D/+^; LSL-Trp53^R172H/+^; Pdx-1-**C**re
MAPK: mitogen-activated protein kinase
NSCLC: non-small cell lung cancer
PDA: pancreatic ductal adenocarcinoma
PDGFR: platelet-derived growth factor receptor
PEG: polyethylene glycol
PEGPH20: pegylated human recombinant PH20 hyaluronidase enzyme
PLN: para-aortic lymph node
PI3K: phosphoinositide 3-kinase
RHAMM: receptor for hyaluronan-mediated motility
rHuPH20: recombinant human PH20 hyaluronidase
RTK: receptor tyrosine kinase
SEM: standard error of the mean
SHAP: serum-derived hyaluronan-associated protein
SPAM-1: sperm adhesion molecule-1
Stab2: stabilin-2
tIFP: tumor interstitial fluid pressure
TGFβR: transforming growth factor beta receptor
TGI: tumor growth inhibition
TLR: toll-like receptor
TNC: tenascin-C
TNF-α: tumor necrosis factor-alpha
TSG-6: tumor necrosis factor-inducible gene 6 protein
UDP: uridine 5' diphosphate
VEGF-A: vascular endothelial growth factor A

## 1. Introduction—The Tumor Stroma

A solid tumor is a highly specialized organ-like structure in disequilibrium with the rest of the body. For the purpose of this review, we have conceptually divided the tumor into two functional parts, malignant cells and the tumor stroma, and will focus this review on the role of the glycosaminoglycan hyaluronan (HA) in the interaction between the tumor stroma and the associated malignant cells. We will also discuss recent preclinical evidence supporting therapeutic targeting of tumor stroma HA via depletion of HA.

The tumor stroma, a supportive framework for malignant cells, consists of various non-malignant cell types and the associated tumor extracellular matrix (ECM), with its multitude of embedded growth factors and protumorigenic elements [1]. Specialized cell types found in the tumor stroma include fibroblasts, myofibroblasts, endothelial cells, pericytes, tumor-associated macrophages and varying numbers of other immune cells [2,3]. More than just a passive bystander to disease progression, the tumor stoma undergoes massive remodeling in response to signals derived from both malignant and host cells, evolving from a barrier to tumor growth to an active contributor of malignant progression [3].

While the communication between the malignant cells and stromal cells may help drive the chronicity of the malignancy, it also makes the tumor stroma an emerging source of targets for the treatment of malignant disease. Indeed, a number of candidate cancer therapeutics have been identified from studies focused on malignant cell:stromal cell interactions (see recent reviews [4,5]). Probably the best known cancer therapeutic to come from these studies is trastuzumab (Herceptin^®^), an antibody inhibitor of the p185^HER2^ receptor tyrosine kinase (RTK). It was discovered in a screen for monoclonal antibodies that reversed the macrophage-resistant phenotype of tumor cells, making them more sensitive to killing by macrophage-elaborated tumor necrosis factor-alpha (TNF-α) [6]. Additional examples of therapeutic targets identified from malignant cell:stromal cell interaction studies include: bevacizumab, which inhibits vascular endothelial growth factor A (VEGF-A), an angiogenesis-related growth factor, preventing stromal vessel formation [7]; vismodegib, a small molecule inhibitor of the hedgehog pathway which targets both tumor stroma and cancer stem cells and has shown very promising results in preclinical studies, and which is currently being tested in several clinical trials of pancreatic adenocarcinoma (PDA) [8]; and TH-302, a hypoxia-activated prodrug which has also been shown to increase the antitumor activity of a broad range of conventional chemotherapy agents in human xenograft models and for which clinical testing is ongoing [9,10]. Similarly, and the focus of this review, the accumulation of HA in the tumor stroma is now recognized as a stromal target for the experimental treatment of malignancies [11,12,13,14], and is the subject of ongoing clinical investigations using an intravenously delivered HA-depleting enzyme, specifically a pegylated human recombinant PH20 hyaluronidase (PEGPH20) [15,16].

## 2. Hyaluronan (HA)

HA, a large, unbranched, non-sulfated glycosaminoglycan composed of repeating disaccharide units of D-glucuronic acid and *N*-acetylglucosamine, is a major constituent of the ECM of most tissues. While simple in composition, HA polymers can reach up to 25,000 disaccharide units, with molecular weights in the 10^7^ Dalton range, and are capable of organizing into complex structures and interacting directly or indirectly with many components of the ECM. The enzymes that produce HA are hyaluronan synthases (HAS). There are three HA synthases (HAS1-3) in mammalian cells, each of which possesses the two glycosyltransferase activities responsible for the transfer of *N*-acetylglucosamine and D-glucuronic acid from their uridine 5'-diphosphate (UDP) precursors to the growing HA chains [17]. The three isoforms are independently regulated and are thought to produce distinct molecular weight forms of HA [18,19,20,21,22]. HAS2 deficiency has been shown to be embryonic lethal, whereas the other synthases may play more subtle roles in development [23,24]. Unlike most other glycosaminoglycans, which are processed and extensively modified in the Golgi, HA is synthesized at the inner face of the plasma membrane and secreted as a linear undecorated polysaccharide directly into the extracellular space. The kinetics controlling HA synthesis *in vivo* are largely unknown but are likely dependent on available cytosolic UDP-monosaccharide concentrations [25]. In malignancy, HAS activity generates large intact linear molecules of HA that are either rapidly incorporated into the ECM surrounding the tumor cells, or retained at the cell surface through HA binding receptors and interacting glycoproteins and proteoglycans. HA may also be tethered to the cell surface by interactions with HAS proteins themselves [26,27,28]. Transgenic studies have shown that while their expression is not itself transforming, HA synthases can cooperate with oncogenes to promote tumor growth and desmoplastic transformation of the tumor stroma [29].

HA degradation is carried out by a family of enzymes called hyaluronidases (HYAL). Genomic analysis reveals that mammals possess multiple hyaluronidase genes, including HYAL-1 through HYAL-5, PH20, and in humans a pseudogene designated HYALP-1 [30]. None of the degradative hyaluronidase enzymes have a lethal phenotype [31,32,33,34,35,36]. HYAL-5 appears to have enzymatic activity redundant to PH20, except for a difference in pH optima, and is not expressed in humans. PH20 is encoded by the sperm adhesion molecule-1 (SPAM-1) gene, and has been studied mostly in the testes where it is localized to the heads of mature sperm. An engineered therapeutic hyaluronidase, a human recombinant form of PH20 (rHuPH20) has been developed, and is approved for use in the local dispersion of co-injected drugs, and is currently being tested in clinical trials to facilitate the subcutaneous delivery of macromolecules [37,38,39].

## 3. The Role of Hyaluronan in the Tumor Stroma

**Figure 1:**
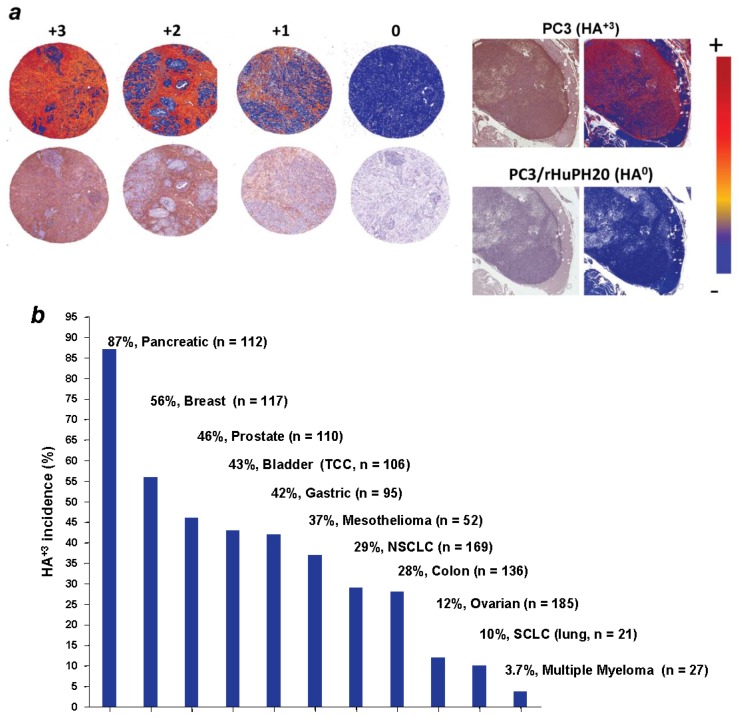
HA accumulation occurs in 87% of pancreatic cancer, and 25−30% of solid tumors overall. (**a**) HA was detected in samples by immunohistochemistry using biotinylated HA binding protein as described in Jacobetz *et al.* [11]. A score of HA^+3^ corresponds to >25% strong positive stain area as compared to the total stain per field. PC3 prostate cancer xenografts were used as a positive control to determine the threshold for positive pixel quantity. Specificity of the staining was confirmed by pretreating a section of same sample with PH20 hyaluronidase in parallel with the test section; (**b**) The percentage of HA^+3^ tumors in different tumor types [11].

Due to its high negative charge, HA is capable of coordinating up to 10,000 times its weight in water, which contributes to its ability to increase interstitial fluid pressure at those sites where it accumulates [40]. Overall, about 25−30% of human tumors overexpress HA, and of special interest for this review, up to 87% of PDAs express high levels of this glycosaminoglycan (Figure 1) [11]. In particular, in tumors that originate from epithelia, which normally express low levels of HA (e.g., breast, prostate, bladder and colorectal cancer), cancer cell-associated HA accumulation is a negative prognostic indicator for cancer patients [41]. The aberrant accumulation of HA in tumors likely occurs via dysregulation of HA synthases and hyaluronidases during disease progression [42,43]. Indeed, it has been suggested that different HAS isoforms might be expressed in different stages of tumor growth to maximize survival of the cancer cells [43]. Whether the constant degradation and resynthesis of HA in tumor foci characterized by high levels of accumulation of this glycosaminoglycan may contribute to the state of immune activation within a tumor is the subject on ongoing research [44].

The ability of HA to imbibe water molecules coupled to its overexpression in the tumor foci contributes to the high interstitial fluid pressure observed in tumors. Indeed, high concentrations of HA in the ECM cause tissue spaces to become hydrated and to swell due to the HA-mediated increase in tissue osmotic pressure [45]. As compared to normal tissues, tumor interstitial fluid pressure (tIFP) can be elevated more than 30-fold, resulting in compression of blood vessels, hypoxia and drug resistance [46]. While overexpression of HA alone in the absence of other cross-linking matrix proteins does not appear to be capable of causing elevated interstitial fluid pressure [47], when sequestered in the tumor stroma together with other ECM components it can significantly contribute to elevated tIFP.

High tIFP may also contribute to metastasis [48]. Several instances of malignant disease that link accumulation of HA with poor prognosis in cancer patients have been reported, and are discussed in detail below. The cross-linking of the ECM by HA not only has the potential to trap protumorigenic growth factors and cytokines, it can also physically interfere in the ability of immune cells to access malignant cells. A more simplistic, but equally intriguing explanation may be that elevated tIFP may in of itself generate a mobility signal for tumor cells to disseminate towards low pressure environments [49].

## 4. The HA Interactome in Cancer

**Figure 2:**
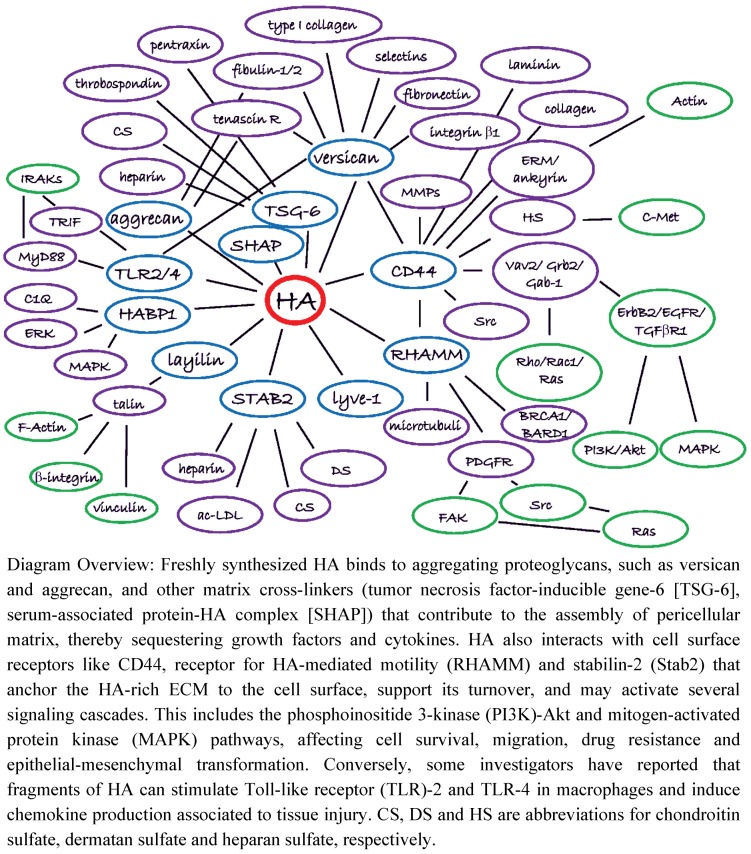
The HA Interactome. HA is produced at the plasma membrane of cancer cells and/or stromal cells by HAS enzymes and is extruded to the extracellular space where it forms a meshwork with its interacting molecules, the hyaladherins, playing multiple complex roles in cell adhesion, motility and proliferation. This diagram emphasizes interactions between HA and its binding molecules, and downstream secondary and tertiary interactions. Although shown as a single interactome in this figure, the indicated interactions might be cell type- and condition-dependent.

HA binds directly to molecules collectively referred to as hyaladherins. These hyaladherins include proteins with diverse molecular functions, such as versican and CD44 [50,51]. Here, we categorize hyaladherins into two groups: (1) matrix proteoglycans and glycoproteins; and (2) cell surface receptors. A summary of all hypothetical HA molecular interactions, the HA interactome, illustrating HA’s binding partners and associated downstream effectors, is presented in Figure 2. Both direct and indirect interactions as reported in the literature are shown, and it is understood that there are varying degrees of evidence to support the interactions shown in Figure 2.

### 4.1. HA Interactions with ECM Components

Matrix proteoglycans and glycoproteins cross-link hydrophilic HA to the ECM, thereby having a major impact on the structure and physicochemical properties of the tissue [52]. Further, this “cross-linked” HA-rich matrix, in interaction with its binding partners, sequesters growth factors and cytokines in the tumor microenvironment which affects the behavior of surrounding malignant and stromal cells [43]. As an example, in PDA, which is characterized by stromal desmoplasia (the formation and development of fibrous tissue) and hypovascularity, stromal accumulation of HA and other matrix components leads to high tIFP, vascular compression and inefficient drug delivery [11,12]. Aggregating proteoglycans such as versican and aggrecan also have an important role in the formation of pericellular HA-rich coats in many cell types that regulate cell proliferation and migration. For instance versican, by binding to type I collagen, tenascin-R, fibulin-1, fibulin-2, fibronectin and fibrillin-1, cross-links these matrix components which facilitates tumor invasion and metastasis [53,54,55,56,57,58,59,60,61,62]. Interestingly, HAS2^−/−^ mice and heart-defect (hdf) mice lacking versican have a similar phenotype: a defect in the formation of cardiac jelly and endocardial cushions. It seems that neither HA nor versican is sufficient alone, but both components are needed for the migration and transformation of endocardial cushion cells during heart development [23,63]. The importance of the HA-versican interaction is further supported by the observation that versican-HA aggregates, but not HA alone, promote angiogenesis and stromal cell recruitment in a mouse model of breast cancer [29].

HA can also be covalently cross-linked to the ECM by SHAP and TSG-6. SHAP is composed of heavy chains of inter-α-inhibitor (IαI) and forms a covalent linkage to HA. Heavy chains are transferred from chondroitin sulfate of IαI onto HA, with TSG-6 as a catalyst and co-factor [64,65]. Elevated SHAP-HA complex levels are found in the serum of cancer patients [66]. TSG-6 is also reported to bind to HA as oligomers, and to form complexes with pentraxin [67] and thrombospondin [68], bringing several HA molecules together.

### 4.2. HA Interactions with Cell Surface Receptors

HA also interacts with cell surface receptors, thereby anchoring the HA-rich ECM to the cell surface. Activation of these HA receptors may likewise stimulate signaling cascades, thereby affecting tumor cell survival, migration, drug resistance and epithelial-mesenchymal transformation. These functions may be mediated by HA synthesis-induced microvilli on tumor and/or stromal cells [69,70]. For instance, microvilli may facilitate HA-induced migration and invasion by providing new attachment sites and possible sites for ECM-degrading enzymes. The microvilli-containing lipid raft microdomains [69] may also serve as a preferred site for multidrug transporters and signaling molecules.

Indirectly, as a result of its interactions with cell surface receptors, HA appears to impact the clustering of RTKs, thereby influencing the constitutive activation of multiple growth-associated receptors [71]. Indeed, several studies have implicated HA in the activation of the human epidermal growth factor (EGF) receptor (HER) family of RTKs via interaction with the transmembrane HA receptor CD44, the major HA cell surface receptor [72]. Although the role of CD44, with its multiple isoforms, in tumor progression appears complex and is not completely understood [73,74], binding of HA clearly induces CD44 clustering and activation. Activated CD44 then directly or indirectly (via adapter proteins) interacts with RTKs and growth-associated receptors (transforming growth factor beta receptor [TGFβR], platelet-derived growth factor receptor [PDGFR] and Erb [HER] family receptors) as well as non-receptor kinases, such Src or Ras family GTPases. This may activate downstream signaling cascades, especially the PI3K/Akt and MAPK pathways [72,75,76,77]. Finally, CD44 also interacts with actin cytoskeleton and affects cell shape and motility by binding to ankyrin [78] and ezrin/radixin/moesin (ERM) proteins [79].

Another HA cell surface receptor, RHAMM, is found in most mammalian cells and its expression is increased in tissue injury and cancer. In addition to its localization in cytoplasm and nucleus, RHAMM can be found at the cell surface, though it does not contain a membrane-spanning domain, nor does its mRNA sequence encode a signal sequence for secretion from the endoplasmic reticulum/Golgi complex. It has also been reported to attach to the plasma membrane via glycosylphosphatidylinositol (GPI) anchor, and can be shed or secreted from cells [80]. Binding of HA to RHAMM at the cell surface, or putatively in the cell cytosol, activates several signaling cascades via Src and Ras [81,82,83], and intracellular RHAMM can bind to microtubules of the mitotic spindle to regulate mitosis [84]. Intracellular RHAMM has also been suggested to have scaffolding functions to control the activity and targeting of extracellular signal-regulated kinase (ERK)1/2 to tubulin [85]. While bound to BRCA1, RHAMM regulates the normal development of breast epithelial cells and may influence the risk of breast cancer [86,87,88]. RHAMM can also bind directly to CD44 at the plasma membrane, and activate CD44 signaling through ERK1/2, potentially enhancing cancer cell migration [89], and indicating the collaboration of different receptors to activate signaling pathways.

As highlighted previously, HA is a polymer of disaccharides (D-glucuronic acid and *N*-acetylglucosamine), which can vary in length from small HA oligosaccharides to 2,000–25,000 disaccharide unit polymers thought to normally dominate the ECM of tissues [24]. Accordingly, evidence has suggested that low molecular weight HA polymers of different sizes display unique, size-specific receptor binding activities in the stromal milieu (see Section 9.2) [90,91,92,93,94,95]. Small HA fragments, for instance, have been reported to interact with TLR-2 and TLR-4 in macrophages, inducing chemokine and cytokine production associated with tissue injury [90]. Small HA fragments have also been shown to enhance migration of melanoma cells in a TLR-4-dependent manner [91], and larger HA oligosaccharides induce the maturation of dendritic cells via TLR-4 [92]. Of note, versican also binds to TLR-2 and acts as an agonist for the receptor to activate tumor-infiltrating myeloid cells by increasing production of cytokines, like TNF-α, to promote tumor metastasis [96]. Additionally, both HA fragments and low molecular weight HA (15–40 kDa) have been shown to have the capacity to bind RHAMM directly [93,94,95], and investigators have reported that in fibrosarcoma cells low molecular weight HA increases adhesion via RHAMM-mediated focal adhesion kinase (FAK) and ERK1/2 signaling [93], while in endothelial cells HA fragments bind to RHAMM and stimulate tyrosine phosphorylation of p125FAK, paxillin and p42/44 ERK associated with induction of adhesion and proliferation [95].

Recent findings in the role of non-traditional hyaladherins in tumor progression have highlighted the importance of HA-receptor interactions in cancer biology. Inhibition of both layilin and Stab2 suppresses tumor metastasis in animal models [97,98], while HA binding protein 1 (HABP1) promotes motility of melanoma cells and tumor growth [99]. Furthermore, HABP1 is suggested to be a key regulator for lamellipodia formation and cancer metastasis [100]. Low levels of tumor HABP1 have been associated with longer survival rates in breast cancer patients [99].

It is clear from studies to date that disruption of HA-rich tumor stroma by depletion of this centrally active molecule has the potential to lead to a significant impact on tumor behavior by inducing a complete reorganization of the ECM and collapse of the tumor stroma. Enzymatic depletion of HA from tumors, using the hyaluronidase PEGPH20, remodels the tumor microenvironment by decreasing at least collagen 1 and 5 and tenascin-C content [12,13] and leads to tumor growth inhibition associated with a reduction in DNA synthesis. In preclinical models, removal of HA also leads to normalized tIFP, decompression of vasculature, more efficient drug delivery, and increased survival [11,12,14].

## 5. Significance of HA Accumulation in Cancer and the Early History of Hyaluronidase Therapies

Literature reports from the early 1950s have associated overproduction of HA with malignancy [101,102]. Later work [103,104,105] provided preliminary evidence that the spreading factor activity of hyaluronidase enhanced clinical efficacy of chemotherapy in doses up to 200,000 U/day. In addition, previous preclinical studies [106,107,108] have attributed chemopotentiation by hyaluronidase to a reduction in tIFP, which facilitates enhanced drug penetration into HA-depleted tumors. Increased production of HA in tumor cells, via ectopic HAS expression, has been shown to induce microvillus structures, suppress contact inhibition, and increase tumor growth rates *in vivo* [13,29,69,109,110]. Conversely, reduction of tumor HA levels in preclinical studies, either by addition of hyaluronidase or by inhibition of HAS activity, has been reported to reduce *in vitro* tumor cell proliferation, motility and invasion, and to reduce the growth of implanted tumors [13,14,21,111,112,113,114,115]. Similarly, inhibition of HA synthesis by 4-methylumbelliferone has been shown to inhibit cancer cell adhesion, breast cancer, and melanoma cell invasion and melanoma metastasis *in vivo* [116,117,118,119]. Recent work which helps to define the utility of PEGPH20 in the treatment of human cancer will be the subject of discussion later in this review.

Further exploration of the role of HA accumulation in tumor progression in patients has included clinical work with several solid and hematopoietic tumors [41,120]. In breast adenocarcinoma, five-year survival deteriorated as a function of increasing stromal HA levels; for low, moderate, and high HA levels, respectively, the five-year overall survival was 45%, 39%, and 26% (*p* = 0.002) and recurrence-free survival was 66%, 56%, and 40% (*p* = 0.008). The presence of HA-positive carcinoma cells correlated significantly with axillary lymph node positivity and poor differentiation. The five-year overall survival of patients exhibiting HA-positive carcinoma cells was significantly lower compared to patients without HA-positive carcinoma cells (54% *versus* 81% respectively, *p* = 0.01) [120].

In gastric carcinoma, the HA profile of 215 Stage I–IV gastric carcinoma patients was examined. A high proportion of HA-positive cells was found that was significantly associated with deep tumor invasion, nodal metastasis, positive lymphatic invasion, poor differentiation grade, as well as inferior prognosis in univariate survival analysis. Forty-four percent of the tumors evaluated had an HA labeling index of 30–100% HA-positive cells [121]. In colorectal carcinomas, the cellular association of HA to overall survival and recurrence-free survival in 202 colorectal carcinoma samples was followed for a mean of 14 years. Both high HA intensity and labeling indices were frequently found and significantly associated with poorer overall survival, shorter recurrence-free survival, and elevated Dukes classification for 187 evaluable patients [122].

HA levels were studied in 309 epithelial ovarian cancers and 45 matched metastatic lesions. While in 73% (227 of 309) of the cases the fraction of HA-positive cancer cells was <10%, high stromal HA levels were significantly correlated with poor differentiation, serous histologic type, advanced stage, and large primary residual tumors [123]. Accumulation of HA is similarly reported to predict worsened outcomes in prostate, bladder and squamous cell carcinoma-type non-small cell lung cancers (NSCLC) [124,125,126]. Recent data suggest that pancreatic cancer has a high frequency of HA overexpression at about 87% (Figure 1) [11]. In other complementary studies, the expression of HA receptors CD44 and RHAMM have been found to predict more aggressive disease in hematologic neoplasias [127,128] and colon cancer [129]. Both of these receptors have been shown to be dysregulated by inactivation of the P53 tumor suppressor gene [130,131].

While early clinical investigations of hyaluronidase in cancer patients [103,104,105] demonstrated potential therapeutic activity, these studies were curtailed because of concerns of potential anaphylactic responses to the foreign proteins that are found in hyaluronidase products from animal sources and a remarkably short plasma half-life. In order to pursue the potential therapeutic utility of the hyaluronidases in cancer therapy, a pegylated form of recombinant human PH20 hyaluronidase was developed, PEGPH20. Whereas the non-pegylated recombinant human PH20 enzyme, rHuPH20, has a very short serum residence time (t_1/2_ = 2.3 min), the polyethylene glycol (PEG)-modified rHuPH20 (PEGPH20) exhibited an increased *in vivo* half-life in mice of ∼270-fold (t_1/2_ = 10.3 h), making it well suited for subsequent studies evaluating the effect of HA removal and the subsequent stromal remodeling of HA-overexpressing tumors [14].

## 6. Preclinical Proof of Concept for Therapeutic Targeting of HA in the Tumor Stroma

**Figure 3:**
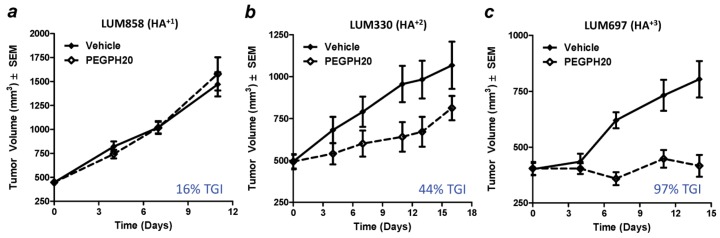
Prospective testing of response to pegylated PH20 hyaluronidase (PEGPH20) as a function of HA phenotype. Squamous cell carcinoma-type NSCLC patient explants were first characterized for HA expression by HA staining [13] using biotinylated HA binding protein and subsequently grown in nude mice to test the hypothesis that the HA^+3^ phenotype (**c**) would respond more robustly to single-agent PEGPH20 than either the HA^+2^ (**b**) or HA^+1^ (**a**) phenotypes. TGI = tumor growth inhibition. Error bars in Figure 3a–c represent SEM of averages of tumor volumes from 10 mice. Reprinted by permission [13]

Characterization of multiple tumor cell lines for HA expression, followed by examination of their response to the exogenous administration of PEGPH20 as transplanted tumors in animal models, demonstrated that tumors which express elevated levels of HA *in vivo* were more sensitive to the antitumor effects of PEGPH20 [13,14]. To test whether higher HA-expressing tumors are more sensitive to HA depletion, a set of NSCLC patient explants was analyzed for HA content using a semiquantitative scoring method that identified squamous cell lung cancers with HA^+1^ (low), HA^+2^ (medium), or HA^+3^ (high) phenotypes. Patient explants were used for this study in order to best imitate the genetic heterogeneity of tumors *in situ*, while hopefully maintaining some stromal elements that might be important to HA production [132] and response to PEGPH20. The results showed dramatic differences in the degree of response, depending upon the degree of tumor-associated HA (Figure 3) [13]. Tumor growth inhibition responses were 97% for HA^+3^ (LUM697), 44% for HA^+2^ (LUM330), and 16% for HA^+1^ (LUM858) NSCLC patient explants grown in nude mice. This suggests that in clinical trials testing stromal HA depletion using PEGPH20, pre-selection for an HA^+3^ phenotype may enhance the response rate in treated patients.

## 7. Enzymatic Targeting of Tumor HA

The exogenous administration of hyaluronidase has been shown to enzymatically degrade HA both *in vitro* and *in vivo*, removing HA pericellular matrices from cultured cancer cells [13,14,69] and depleting tumor HA in preclinical animal models [11,12,13,14,111].

**Figure 4:**
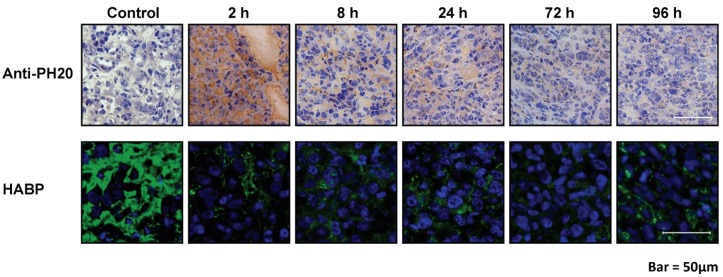
Impact of intravenous PEGPH20 treatment on tumor-associated HA in prostate cancer xenografts. Assessment of PEGPH20 and HA levels in PC3 xenografts harvested at 2, 8, 24, 72, and 96 h following intravenous administration of PEGPH20 (15 mg/kg). PEGPH20 and HA levels were assessed using an antibody to rHuPH20 and HABP, respectively. PEGPH20-mediated HA removal was sustained for >72 h, with a gradual return of HA signal by ∼10 days (data not shown). The tissue areas selected for representation are slightly different between the anti-PH20 and HABP sections. Experimental details are given in Thompson *et al.* [14]. Reprinted by permission [14].

In studies of PC3 prostate cancer xenograft tumors, administration of a single intravenous bolus dose of PEGPH20 effectively removed HA from the tumor stroma within 2 h following administration, compared with tumor-bearing mice treated with vehicle alone, and the HA removal was concomitant with the appearance of PEGPH20 in the tumor (Figure 4). Further, following enzymatic treatment with PEGPH20, tumor HA was essentially absent for 3 days, gradually appearing by Day 10 [14].

**Figure 5:**
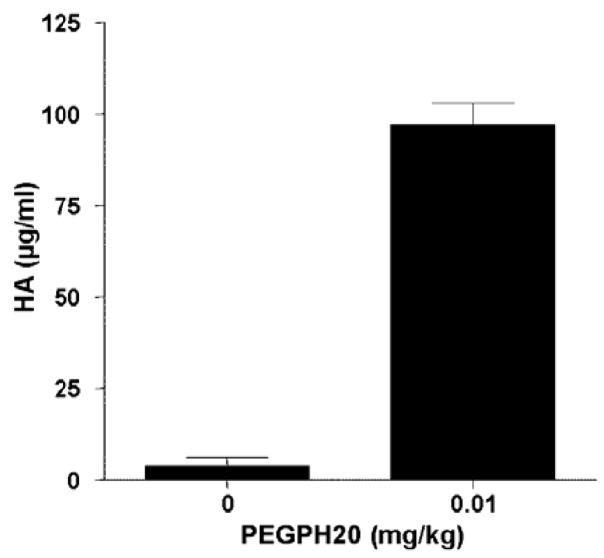
HA concentration in serum following *in vivo* treatment with PEGPH20 is a pharmacodynamic marker of hyaluronidase activity. Balb/c mice carrying 4T1 mammary tumors at volume of ~400 mm^3^ were injected intravenously with vehicle or 10 µg/kg PEGPH20. Plasma was collected 24 h post-PEGPH20 administration and HA levels were analyzed using Hyaluronan DuoSet kit (R&D Systems). The HA level was significantly elevated in the mice treated with PEGPH20 (*p* < 0.001). Error bars in the figure represent SEM of averages from five mice.

The degraded HA immediately has access to extracellular spaces and the circulatory system, including the lymphatics. The appearance of HA in the blood is a convenient pharmacodynamic marker for the hyaluronidase activity of PEGPH20 in animal models and patients (Figure 5), and can assist in monitoring HA degradation *in vivo*.

**Figure 6:**
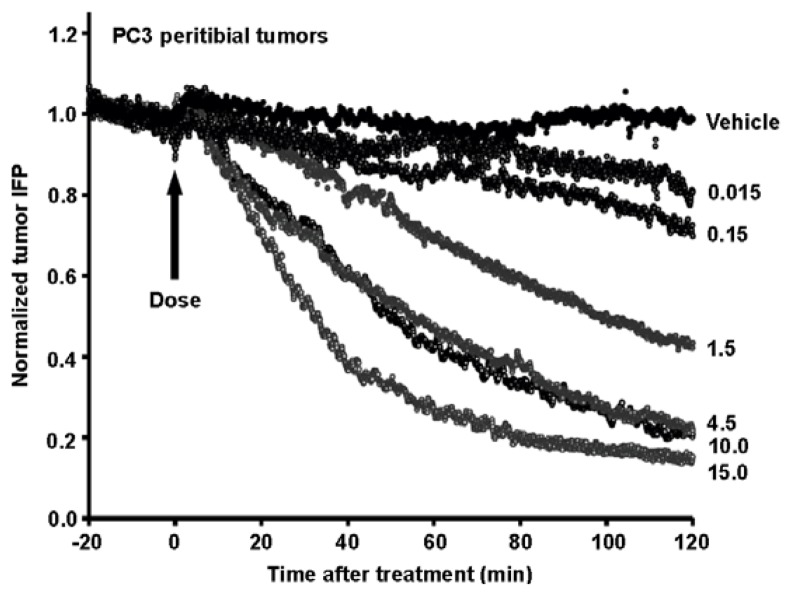
Dose-dependent effect of PEGPH20 *in vivo* on tIFP in PC3 peritibial tumors. Mice were treated intravenously with 0–15 mg/kg PEGPH20 (doses in mg/kg are shown on the right) and tumor IFP was measured over a 2 h period post-administration. Experimental details are given in Thompson *et al.* [14]. Reprinted by permission [14].

Recently, several papers have been published which shed light on the mechanism of antitumor activity mediated by the exogenous addition of PEGPH20 [11,12,13,14], and these provide additional rationale for HA-depleting reagents in the treatment of cancer. At the macrophysiological level, the hyaluronidase PEGPH20 induces massive structural changes in the tumor. These changes are translated into altered gene expression patterns. The most striking biomechanical impact of PEGPH20-mediated HA depletion from an HA^+3^ tumor is rapid loss of tIFP (Figure 6) [14]. Depending on dose and time, interstitial fluid pressures comparable to normal tissue can be achieved *in vivo* within 2 h of treatment. The reduction in tIFP leads to expansion of tumor blood vessels, and vascular re-perfusion [14]. Perhaps paradoxically, this increased tumor perfusion is associated with a decrease in DNA synthesis [13].

**Figure 7:**
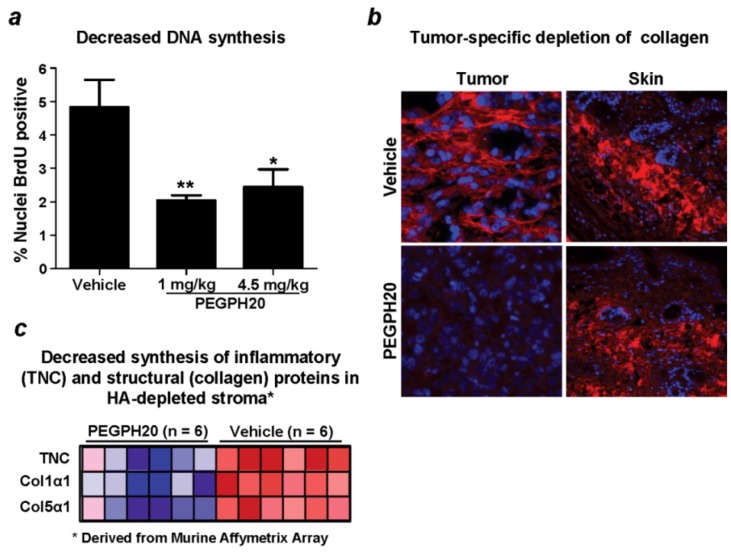
Depletion of HA from the ECM results in decreased DNA synthesis and molecular remodeling of the tumor. Antitumor and stromal-depleting effects are observed after treatment of PC3 (HA^+3^) xenografts with PEGPH20. These include reduced tumor DNA synthesis (**a**); tumor-specific depletion of collagen (**b**); and decreased synthesis of stromal proteins (tenascin C, collagens 1 and 5) (**c**); Experimental details are described in Jiang *et al.* [13]. Error bars represent SEM of averages from six mice. Reprinted by permission [13].

While there are numerous sequelae to these initial changes induced by PEGPH20 alone, it is now clear that these changes alone can result in inhibition of tumor cell DNA synthesis, and reduction in the expression of collagen (collagen 1-alpha-1 [Col1α1]; collagen 5-alpha-1 [Col5α1]) and tenascin-C (TNC) (Figure 7) [13], none of which directly interacts with HA. Thus, depletion of HA alone appears sufficient to induce broad remodeling of the tumor stroma. Depletion of HA, collagen and other stromal components from the ECM has raised the question of whether these changes can impact the metastatic behavior of the malignant cells resident within the stroma.

**Figure 8:**
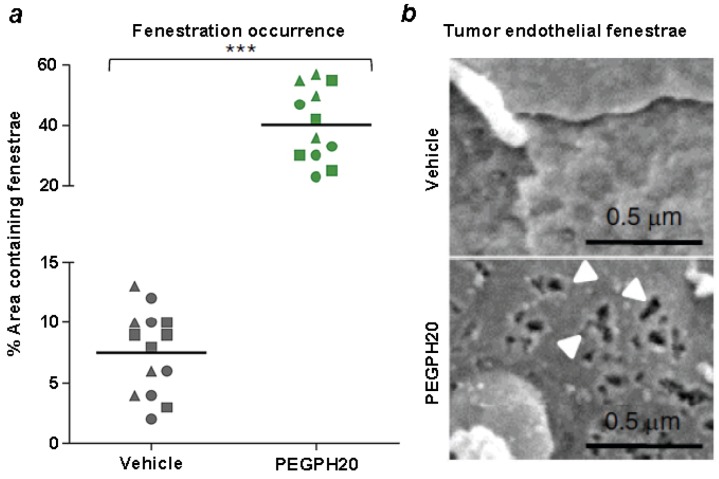
Induction of endothelial fenestrae as a result of treatment with PEGPH20. (**a**) The frequency of vascular fenestrae in KPC tumors was estimated using scanning electron microscopy and quantified in 4 random vessels in 3 mice per cohort; (**b**) Visualization of fenestrae from vehicle-treated and PEGPH20-treated KPC mice with PDA by scanning electron microscopy (n = 4 for each cohort). Fenestrations were found in pancreatic blood vessels of PEGPH20-treated tumors. PEGPH20 did not induce formation of fenestrae in normal pancreatic blood vessels of the control (PC) mouse. Experimental details are described in Jacobetz *et al.* [11]. Reprinted by permission [11].

In addition to the xenograft models which provided initial proof of concept for the antitumor activity of PEGPH20, a novel model of pancreatic cancer using a genetically engineered mouse model, called the KPC (LSL-Kras^G12D/+^; LSL-Trp53^R172H/+^; Pdx-1-Cre) mouse has recently been employed [133]. The advantage of this model is that it develops autochthonous tumors that appear to mimic structural aspects and clinical symptoms of human PDA [134]. PDAs that occur in this model commonly accumulate HA and express high tIFP [11,12]. Treatment of KPC tumors with PEGPH20 depletes tumors of HA, leads to the expansion of intratumoral blood vessels, and, similarly to xenograft models [11,12,14], increases delivery of chemotherapy (doxorubicin, gemcitabine) to the tumor [11]. While similar effects have been reported by other agents that target stromal targets, e.g., the hedgehog pathway [134], the mechanism by which this occurs has not been defined. In the case of PEGPH20, the rapid expansion of blood vessels as HA is depleted leads to ultrastructural changes in tumor endothelium, characterized in part as a significant increase in endothelial fenestrae (Figure 8) [11]. No similar changes in the non-tumor-associated endothelium are observed [11], which suggests that increased fenestrae result from the abrupt change in vessel structure once tIFP is reduced and blood vessels rapidly expand.

## 8. Combining Therapeutic Targeting of Tumor HA with Chemotherapy

As a single agent, hyaluronidases routinely show therapeutic efficacy in preclinical tumor xenografts that accumulate HA [13,14,111]. Shuster and co-workers reported that purified testicular hyaluronidase suppresses tumor growth of breast cancer xenografts [111], while removal of HA using PEGPH20 has been efficacious in mouse xenograft models of human prostate, breast, lung and pancreatic cancer and in human NSCLC explant models (see Figure 3) [11,13,14]. It is likely that this antitumor effect is related to the major structural alterations induced in the tumor (stroma, including vasculature) resulting from stromal remodeling, and subsequent changes in gene expression. These structural changes, especially vascular expansion and formation of fenestrae, which occur only in the impacted tumor stroma and not in normal tissues, are among the alterations observed in tumor vasculature following treatment with PEGPH20. This outcome allows for the possibility of synergistic antitumor activity of hyaluronidases and chemotherapy. Hyaluronidase has been shown to enhance antitumor activity of adriamycin and vinblastine in breast cancer and melanoma mouse models, respectively [106,135]. There are also early clinical studies reporting that co-treatment of hyaluronidase and chemotherapy or radiation therapy added efficacy of the treatment compared to monotherapy in Kaposi’s sarcoma and malignant pediatric tumors [103,136]. On the other hand, in a large randomized study of high-grade astrocytomas, hyaluronidase failed to show a synergstic effect with chemotherapy or radiation therapy upon survival, which was speculated to be due to the intrinsic resistance of high grade astrocytomas to chemotherapeutic agents [104].

Enhanced activity of chemotherapy by co-treatment with PEGPH20 was initially shown in transplantable mouse tumor models [14]. Synergistic activity with the combination of PEGPH20 with chemotherapy was also observed in the transgenic mouse model (KPC) of PDA (Table 1) [11,12]. In this model, PEGPH20 administration (4.5 mg/kg; every 3 days) had no significant effect compared to vehicle alone (~10 days); there was a 43% increase in survival time when animals were treated with gemcitabine (15 days) (administered 30 min post-PEGPH20), and an almost three-fold increase in survival time when animals were treated with a combination of PEGPH20 and gemcitabine (28.5 days) [11].

**Table 1:** Synergistic increase in survival by co-treatment of transgenic mouse model of PDA with PEGPH20 and gemcitabine [11].

Treatment ^1^	n	Median Survival (days)	Increase in Survival ^2^ (%)
Vehicle	7	10.5	0
Gemcitabine	11	15.0	43
PEGPH20	10	9.0	0
Gemcitabine + PEGPH20	11	28.5	271

^1^ Methods are described in Jacobetz *et al.* [11]; ^2^ Gemcitabine *versus* combination of gemcitabine and PEGPH20 (*p* = 0.002).

## 9. Endogenous Hyaluronidases and HA Oligosaccharides as Tumor Promotors or Suppressors

### 9.1. Endogenous Hyaluronidase Activity

Although there is growing evidence that exogenous hyaluronidase administration displays significant antitumor activity in HA-overexpressing tumors, local endogenous hyaluronidase expression, within the tumor milieu itself, has been shown to act as a tumor promoter. In preclinical studies, for instance, overexpression of HYAL2 has been observed to stimulate tumor growth in a mouse model of astrocytoma [137], and Tan and co-workers have reported that HYAL1 overexpression promotes tumor growth and angiogenesis in a breast cancer xenograft model [138]. In bladder and prostate cancer cells, HYAL1 seems to be the major tumor-derived hyaluronidase [139,140] and inhibition of hyaluronidase activity has been shown to suppress LNCaP-AI prostate tumor growth in a xenograft mouse model [141].

Clinically, hyaluronidase activity has been shown to be increased in tumor tissues and/or the serum from prostate, bladder, head and neck, metastatic breast and metastatic brain cancers [140,141,142,143,144,145,146]. Elevated hyaluronidase activity has also been observed in urine from patients with intermediate- or high-grade bladder cancer [147], in urine from children with Wilms tumors [148], and in the saliva of patients with head and neck squamous cell carcinoma [144]. Conversely, in patients with ovarian cancer, Nykopp and colleagues reported that downregulation of HYAL1, with the concomitant decrease in hyaluronidase activity, is correlated with elevated tumor HA content [149].

Lokeshwar and Selzer suggested that whether hyaluronidase functions as a tumor promotor or suppressor might be hyaluronidase concentration dependent [150], as levels that exceed the endogenous concentrations found in tumor tissues (100 milliunits/10^6^ cells) have been shown to reduce tumor growth via the induction of apoptosis [13,14,111,151,152] while lower levels of hyaluronidase present in genitourinary tumors, for instance, promote tumor growth [150].

### 9.2. HA Oligosaccharides in Cancer

Both the exogenous administration of hyaluronidase and the expression of endogenous hyaluronidase within a tumor can result in the generation of HA fragments of various lengths. Lower molecular weight HA polymers and HA oligosaccharides have been reported to have biological activities not associated or opposite than ones of the high molecular weight HA [24,153,154]. Several studies have implicated that HA oligosaccharides promote angiogenesis while high molecular weight HA is anti-angiogenic [154,155]. Short HA oligosaccharides stimulate angiogenesis in both *in vitro* and *in vivo* chick chorioallantoic membrane assays [155], and have been shown to induce proliferation, tube formation and collagen production of endothelial cells [156,157,158]. Furthermore, HA fragments of 3–25 disaccharides have been reported to be present in high-grade prostate cancer tissue and in the urine of patients with highly invasive bladder cancer [140,159]. On the other hand, HA oligosaccharides inhibit tumor growth in xenograft models of melanoma, malignant peripheral nerve sheath tumor, ovarian cancer, lung cancer and breast cancer, as well as in mouse models of lung and bone metastasis [160,161,162,163,164,165]. Toole and co-workers have shown that HA oligosaccharides suppress the PI3K/Akt cell survival signaling pathway and reverse drug resistance in cancer cells via CD44 receptor [165,166].

The physiologic effects of HA oligosaccharides likely depends on the size and local concentration of the HA fragments in the tumor [167]. Of note, there has been no evidence of new vessel formation in preclinical studies conducted with PEGPH20 [11,12]. One hypothetical explanation, in agreement with the premise that hyaluronidases function as tumor promotors or suppressors depending on local tumor hyaluronidase concentration [150], is that intravenous doses of long-acting PEGPH20 generate predominately small HA fragments which do not stimulate angiogenesis. Interestingly, Itano and Kimata have suggested that what controls the angiogenic response to HA is a balance between regulatory high molecular weight HA and effector HA oligosaccharides [43]. Ultimately, the role of HA fragments as suppressors or promoters of tumor growth seems to be a very complex phenomenon that depends upon an interplay between the tumor cells, HA receptors, associated HA binding proteins and a variety of angiogenic and other confounding factors that may be unique for every individual tumor [167].

## 10. Therapeutic Targeting of Tumor HA and Metastasis

**Figure 9:**
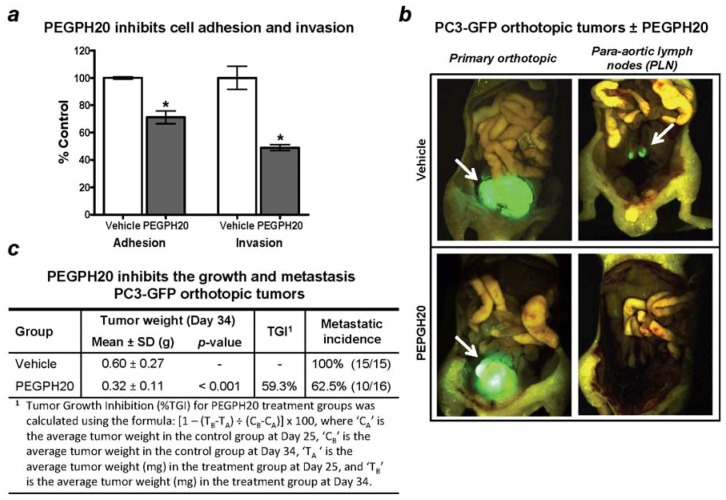
PEGPH20 effects on metastasis. PEGPH20-mediated HA removal inhibits cell adhesion, cell invasion and reduces metastatic incidence. (**a**) PEGPH20-mediated *in vitro* pericellular HA removal reduced cell adhesion by 29% (*p* < 0.001) compared to vehicle alone and reduced the number of PC3 cells invading downward through invasion chambers toward the chemoattractant EGF by >50%, relative to control (*p* < 0.001). Cell adhesion was evaluated as per Cos *et al.* [169], with modifications. Briefly, PC3 cells in culture were harvested, counted, and divided into two groups: growth medium plus 0.1% bovine serum albumin (BSA), and growth medium plus 0.1% BSA and 1,000 U/mL PEGPH20. Both suspensions were then incubated at 37°C for 1h. Cells were subsequently seeded (1.5 × 10^5^ cells/well, n = 5 wells/treatment) onto Matrigel-coated plates and allowed to adhere for 1h at 37°C. At 1h, non-adherent cells were gently removed by washing, and attached cells were then harvested, counted, and expressed as percent relative to control cell adhesion ± SEM. For cell invasion, PC3 cells were incubated with vehicle alone or PEGPH20 (1,000 U/mL) and seeded onto the upper chamber of invasion chambers with either vehicle buffer, or in the case of the PEGPH20-treated cells, additional PEGPH20 to prevent the re-synthesis of HA-rich pericellular matrices. The number of cells invading downward through Matrigel was expressed as percent relative to control cell invasion ± SEM; (**b**) Representative images of PC3-GFP primary orthotopic tumors, and metastatic PLNs, visualized after removal of the primary tumors; (**c**) PEGPH20 treatment (4.5mg/kg; q3d × 3), starting at Day 25, inhibited tumor growth of the primary tumor by >59% within 9 days (by Day 34). Further, the incidence of metastatic PLN tumors was reduced in PEGPH20-treated animals by ~38%, when compared to vehicle-treated animals (100% incidence in vehicle-treated *versus* 62.5% incidence in PEGPH20-treated animals, respectively, *p* < 0.05).

Given the observations that endogenous hyaluronidase expression can act as a tumor promoter (Section 9.1), we aimed to evaluate whether the enzymatic removal of tumoral HA from HA-rich tumors promotes or inhibits metastasis using the highly metastatic human hormone-refractory prostate cancer cell line PC3 [168] which expresses high levels of HA both *in vitro* and *in vivo* [14]. First, to assess *in vitro* whether PEGPH20-mediated removal of extracellular HA influences the attachment (adhesion) of cells to basement membrane, potentially by exposing previously HA-hidden cell adhesion receptors such as integrins, or similarly, the invasion of cells, we used established cell adhesion [169] and Matrigel™ cell invasion assays. PEGPH20 treatment, at a concentration previously shown to completely remove all extracellular HA (1,000 U/mL) [14], reduced cell adhesion by 29% compared to vehicle alone (Figure 9a, left panel); whereas the number of PC3 cells invading downward through the Matrigel toward the chemoattractant EGF was reduced by >50% in cells treated with PEGPH20 compared to vehicle (Figure 9a, right panel). These *in vitro* results indicate that HA depletion may result in anti-metastatic activity, as suggested by recent laminar flow assays which demonstrated that blockade of HA-CD44 interaction by CD44 antibody reduced tumor cell adhesion to HA in several CD44-expressing cancer cell lines [170].

Orthotopic implantation of human prostate cancer PC3 cells into nude mice was used to evaluate the effect of PEGPH20 on metastasis *in vivo*. This model has been shown to lead to the spontaneous formation of adjacent para-aortic lymph node (PLN) metastases [171], and is known to reproducibly generate PLN metastases in 100% of PC3 tumor-bearing mice within 43 days [168]. Accordingly, PC3 cells expressing the enhanced green fluorescence protein (GFP) were injected into the left lobe of prostates. When tumors reached approximately 100 mg (Day 25), the absence of PLN metastasis was confirmed in satellite animals and mice were staged into two treatment groups, vehicle control or PEGPH20. Although it is ideal to remove the primary tumor in most orthotopic spontaneous metastasis models, physical proximity of prostate tumors to the urethra and bladder makes this difficult in mice; additionally, Stephenson and colleagues [171] have previously demonstrated that orchiectomy has little effect on metastasis in this orthotopic model. Three doses of PEGPH20 (4.5 mg/kg; q3d × 3) inhibited tumor growth of the primary tumor by >59% within 9 days, in agreement with our earlier work which showed that higher doses of PEGPH20 reduced tumor growth in peritibially-implanted prostate PC3 cells by up to 70% by Day 27 [14]. At study conclusion (Day 34), primary tumor and metastasis to prostate-adjacent PLNs were evaluated (Figure 9b,c). Primary tumor growth was inhibited by >59% within 9 days (Figure 9b, left panel).

Metastasis occurred in 100% (15 of 15) of vehicle-treated animals; whereas only 62.5% (10 of 16) of the PEGPH20-treated animals were positive for PLN metastatic lesions (Figure 9b, right panel; Figure 9c). Similarly, in the KPC model of PDA, combination treatment of PEGPH20 and gemcitabine decreased metastatic tumor burden compared to gemcitabine monotherapy [12]. Taken together, these studies suggest that PEGPH20-mediated HA removal, following exogenous administration, reduces metastatic incidence. This is further supported by the finding that HA fragments, similar to degradation products of PEGPH20 (Figure 5), decrease metastasis to bone in a mouse bone metastasis model of breast cancer [160].

## 11. Conclusions

In recent years, with the increased recognition of the role of the tumor microenvironment in disease progression, stromal components of the tumor have emerged as attractive targets for therapeutic intervention. One such emerging target is the large glycosaminoglycan HA, a major constituent of the ECM of most tissues, and a molecule which accumulates in many solid tumors [41]. HA actively interacts with its binding molecules, the hyaladherins, which consist of ECM proteoglycans and glycoproteins, such as versican, and cell surface receptors, such as CD44 and RHAMM [29,77,80,172]. The outcome of these interactions, and the associated downstream effectors, the biological complexity of which can be graphically illustrated as a HA interactome, highlight HA’s complex role in tumor cell adhesion, motility, proliferation and invasion [26,42,43,72].

Accordingly, enzymatic depletion of HA can significantly impact tumor behavior by inducing a complete reorganization of the ECM, and in preclinical studies, intravenous administration of a pegylated hyaluronidase is associated with decreased tIFP, expansion of tumor blood vessels, increased delivery of chemotherapeutics, tumor growth suppression and improved survival [11,12,13,14]. The anti-tumor effects observed following the systemic administration of hyaluronidases to HA-overexpressing tumors appears to be in contrast to studies where local endogenous hyaluronidase, secreted within the tumor itself, acts predominantly as a tumor promoter [150,152]. These contradictory findings may be a hyaluronidase “concentration-dependent phenomenon”, with tumor growth inhibition only observed following treatment at the pharmacological levels seen with exogenous administration [150]. Similarly, HA fragments have been shown to both suppress and stimulate tumor progression, and this likely depends on the size and local concentration of the HA fragments in the tumor (*i.e*., the balance between high molecular weight HA and smaller HA fragments) [43,167] which can be regulated by HA synthases and/or the local tumor hyaluronidase concentration [150,167].

In the future, further knowledge about the biology of the HA interactome, as well as the tumor stroma as a whole, and, furthermore, a more complete definition of the important elements of the stroma that dictate aggressive malignancy, will enable new cancer therapies to emerge.
